# The Use of the Drospirenone 4 mg-Only Pill in Overweight and Obese Women with Polyendocrine Metabolic Ovarian Syndrome: A Retrospective Cohort Study

**DOI:** 10.3390/jcm15197701

**Published:** 2026-10-05

**Authors:** Klara Hofer, Selina Ebner, Rodrig Marculescu, Magdalena Boegl, Peter Frigo, Robert Krysiak, Johannes Ott

**Affiliations:** 1 Clinical Division of Gynecological Endocrinology and Reproductive Medicine, Medical University of Vienna, Spitalgasse 23, A-1090 Vienna, Austria; klara.beitl@meduniwien.ac.at (K.H.);; 2 Department of Laboratory Medicine, Medical University of Vienna, A-1090 Vienna, Austria; 3 Department of Internal Medicine and Clinical Pharmacology, Medical University of Silesia, 40-055 Katowice, Poland

**Keywords:** PMOS, hyperandrogenism, hirsutism, drospirenone, Ferriman–Gallwey score

## Abstract

**Objective**: Polycystic ovary syndrome is frequently associated with hyperandrogenemia-related hirsutism, which creates notable cosmetic concerns for many affected women. Although multiple antiandrogenic treatments exist, many patients remain dissatisfied, and combined oral contraceptives are often contraindicated due to metabolic and cardiovascular risk factors. This study evaluates whether a drospirenone-only pill improves hormonal profiles and hirsutism in PMOS compared to cyclic dydrogesterone. **Methods**: This retrospective cohort study assessed hyperandrogenemia and hirsutism using the modified Ferriman–Gallwey score in 60 overweight or obese women with PMOS who were treated at the Clinical Division of Gynecologic Endocrinology and Reproductive Medicine of the Medical University of Vienna, Austria. Patients underwent follow-up examinations after three and six months of treatment. Thirty women chose treatment with drospirenone-only and were age-matched with 30 women receiving cyclic dydrogesterone. **Results**: After 3 months, women in the drospirenone group showed significant improvements, including reductions in LH (*p* < 0.001), FSH (*p* < 0.001), testosterone (*p* = 0.001), FAI (*p* < 0.001) and AMH (*p* = 0.005). Ferriman–Gallwey scores were significantly lower after 3 months (*p* < 0.001) and 6 months (*p* = 0.001). At follow-up, the drospirenone group exhibited lower LH (5.4 vs. 8.8 mIU/mL; *p* = 0.004) and lower FAI (2.9 vs. 4.0; *p* = 0.045) compared with controls. Among women with baseline hirsutism (FGS ≥ 8), scores decreased from 14 to 12 (*p* < 0.001) after 3 months and to 10 (*p* = 0.002) after 6 months in the drospirenone group. **Conclusions**: Drospirenone-only treatment resulted in a modest reduction in Ferriman–Gallwey score, with more pronounced improvements observed in women with baseline hirsutism. This was accompanied by hormonal changes, suggesting a potentially beneficial option for women with hyperandrogenic PMOS for whom combined oral contraceptives are not suitable.

## 1. Introduction

Polyendocrine metabolic ovarian syndrome (PMOS) is one of the most common entities in gynecologic endocrinology with an overall prevalence of about 10% when using the Rotterdam criteria, according to a meta-analysis [1]. PMOS is often associated with a significant reduction in quality of life caused by a variety of factors, including hirsutism, anxiety/depression, sleep disorders, changes in body image and much more. Thus, the cosmetic consequences of hyperandrogenism play a major role for affected women [2]. Around 13% of women with PMOS suffer from hirsutism [1]. There are numerous treatment options to mitigate the effects of increased androgens. These include combined oral contraception (COC), spironolactone, cyproterone acetate and finasteride among others [2]. Despite these available tools and the latest international recommendations for diagnosis, counseling and treatment [2], many PMOS patients are dissatisfied with the medical services on offer. This also applies to the therapeutic options available. A recent study found a dissatisfaction rate of around 70% on this issue [3].

The suppression or antagonization of androgen secretion and action, one of the main goals of treatment [4], can be achieved by different mechanisms of action: (i) Orally administered ethinylestradiol leads to an increase in the levels of sex hormone-binding globulin (SHBG). This reduces the free androgen index (FAI). (ii) The secretion of androgens can be decreased by the luteinizing hormone (LH)-lowering effect of progestins with or without ethinylestradiol. (iii) Several progestins exert an antagonistic effect on the androgen receptor. Last but not least, (iv) the 5 alpha-reductase is inhibited by some progestogens which leads to a reduced local effect of androgens. COCs, especially those containing ethinylestradiol and cyproterone acetate/drospirenone/dienogest often provide all four mechanisms of action, resulting in maximum antiandrogenic effect [5]. Unfortunately, COCs often are contraindicated in PMOS women, mainly in the presence of insulin resistance-related comorbidities [6].

Since one is often worried about possible adverse effects of ethinylestradiol, an antiandrogenic progestin-only contraceptive might also help PMOS patients effectively based on a combination of the LH-mediated decrease in testosterone, the antagonistic effect on the androgen receptor, and the local inhibition of the 5 alpha-reductase. Notably, a new drospirenone 4 mg-only pill has been available for several years. Given its contraceptive effectiveness and its overall safety profile [7,8,9], it has already been mentioned that the drospirenone 4 mg-only pill may have theoretical advantages in PMOS [7]. It has already been demonstrated that, under combined oral contraceptives containing drospirenone, the levels of LH, testosterone and dehydroepiandrosterone sulfate (DHEAS) decline, at least in healthy women [10].

In this retrospective cohort study, we aimed to evaluate the PMOS-specific hormonal profile as well as changes in the Ferriman–Gallwey scoring system (FGS) for hirsutism in PMOS patients who had used the drospirenone 4 mg-only pill or cyclical bleeding induction with dydrogesterone only, a progestin that exhibits negligible androgenic or antiandrogenic properties [11,12,13].

## 2. Methods

This retrospective, monocentric cohort study included 60 overweight/obese (BMI ≥ 25 kg/m^2^) women who were diagnosed with PMOS. PMOS was diagnosed according to the revised European Society of Human Reproduction and Embryology (ESHRE) and the American Society for Reproductive Medicine (ASRM) criteria of 2003 [14]. Following the recent international consensus on nomenclature, the term polyendocrine metabolic ovarian syndrome (PMOS), formerly known as polycystic ovary syndrome (PCOS), is used throughout this manuscript [15].

All women were treated at the Clinical Division of Gynecologic Endocrinology and Reproductive Medicine of the Medical University of Vienna, Austria, between January 2021 and July 2024. Patients were seen at baseline and after three and six months of individual treatment.

As part of clinical routine, this included serum parameters as well as clinical parameters. Hirsutism scores were graded according to the modified FGS system, and patients with a score ≥ 8 were defined as hirsute [16].

None of the 60 patients currently wanted to have children. After having been informed about other treatment options (cyclic progesterone/dydrogesterone treatment, spironolactone, finasteride, local treatment options for cosmetic problems, the drospirenone-only pill, and combined oral contraception) and its side effects, 30 patients had decided on drospirenone-only treatment. These women (the “DRSP-group”) were prescribed the drospirenone-only pill (Lyzbet^®^, Exeltis Austria GmbH, Judenplatz 7/Top 2, 1010 Vienna, Austria) for menstrual cycle regulation and mitigation of androgenic symptoms. These women were matched to 30 overweight or obese PMOS patients (1:1 matching for age; the “DYD-group”), who received cyclic dydrogesterone for bleeding induction only. Dydrogesterone was selected as a clinically relevant neutral comparator because it is routinely prescribed for cyclic bleeding induction in women with PMOS and has no known androgenic or antiandrogenic activity [11,13]. Given the retrospective nature of the study, the treatment allocation reflected routine clinical practice rather than prospective assignment. Thus, the DYD-group was not intended to represent an active antiandrogen comparator, but rather a clinical reference group without expected direct effects on hyperandrogenism.

All participants in both treatment groups were of Caucasian ethnicity. Information on previous or ongoing cosmetic treatments for hirsutism (e.g., laser/IPL treatment, etc.) was not systematically available due to the retrospective study design. However, patients were instructed to refrain from hair removal for at least two weeks before the assessment of the modified Ferriman–Gallwey score. Women who had already received any PMOS-specific medication in the previous three months were excluded. Additional exclusion criteria included pregnancy, breastfeeding, known endocrine disorders other than PMOS (e.g., thyroid dysfunction, Cushing’s syndrome) or incomplete clinical or laboratory data.

### 2.1. Parameters Analyzed

The AKIM-software (version 7, SAP Software Solutions Austria, Vienna, Austria; SAP-based patient management system at the Medical University of Vienna) was used for data acquisition. Basic patient information included age and BMI. Polycystic ovarian morphology (PCOM) was assessed using an Aloka Prosound 6 ultrasound machine (Wiener Neudorf, Austria; frequency range 3.0–7.5 MHz). PCOM was defined by a follicle number per ovary (FNPO) > 12 and/or an ovarian volume ≥ 10 cm^3^, and/or an ovarian area ≥ 5.5 cm^2^. This is consistent with international recommendations, including the use of an ultrasound machine with a frequency range less than 8 MHz [17]. The modified FGS was used to quantify hirsutism in PMOS women [16].

**Figure 1:**
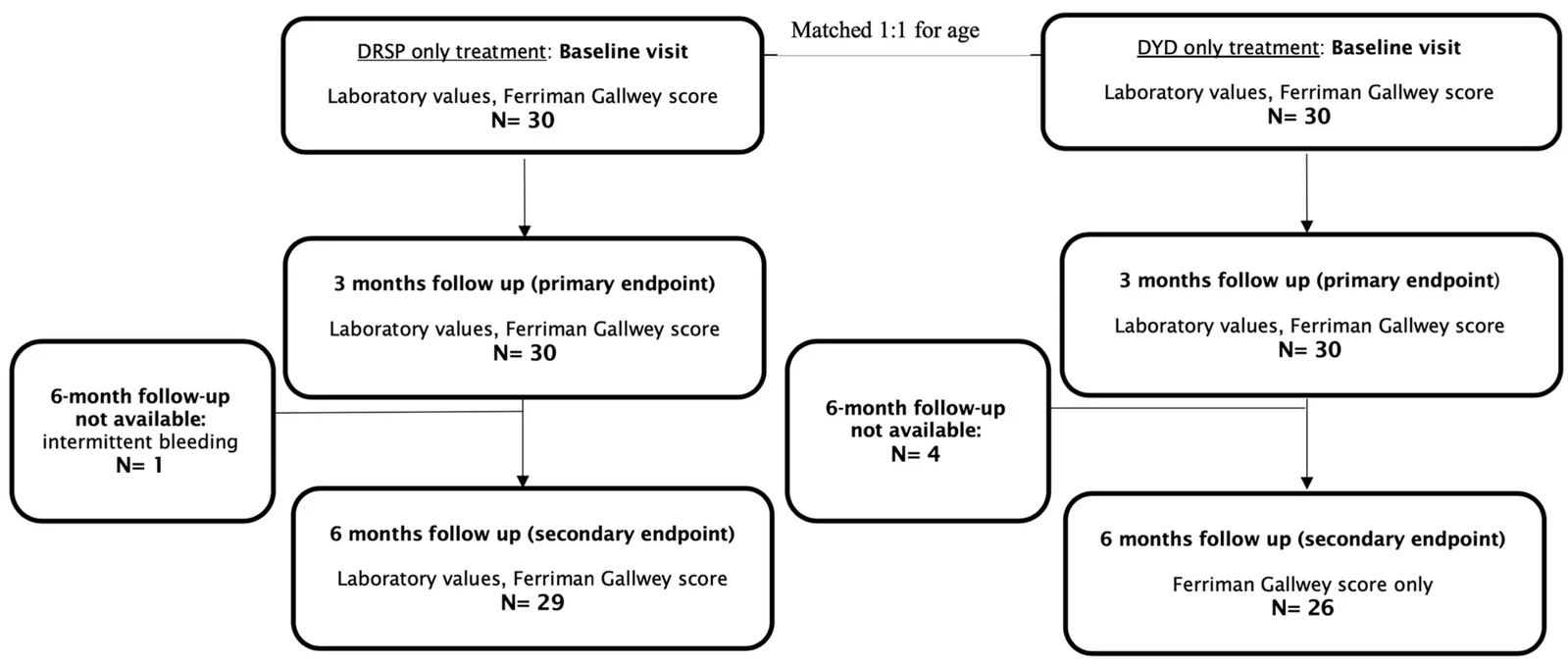
Flowchart. Overweight/obese patients with PMOS were included into the study. PMOS: polyendocrine metabolic ovarian syndrome, DRSP: drospirenone, DYD: dydrogesterone.

Data were collected at the time of initial presentation and subsequently during the follow-up assessments after 3 months of hormonal treatment (primary endpoint). Additionally, in the drospirenone group, parameters were assessed after 6 months (secondary endpoint). For the dydrogesterone group, only FGS data were available at the 6-month follow-up (secondary endpoint) (Figure 1).

As the main outcome parameter, we focused on serum levels of testosterone. Additionally, serum levels of dehydroepiandrosterone sulfate (DHEAS), SHBG, LH, follicle-stimulating hormone (FSH), and anti-mullerian hormone (AMH) were collected. All serum parameters were determined at the Department of Laboratory Medicine, Medical University of Vienna, according to ISO 15189 quality standards [18]. As reported previously [19,20], Cobas electrochemiluminescence immunoassays (ECLIA) were performed on Cobas e 602 analyzers (Roche, Mannheim, Germany) for the determination of serum prolactin, estradiol, FSH, LH, AMH, testosterone, DHEAS, and SHBG. The FAI was calculated as (total testosterone [in ng/mL] × 3.47/SHBG [in nmol/L]) × 100 [21]. The HOMA-IR (Homeostatic Model Assessment of Insulin Resistance), calculated as HOMA-IR = insulin (mU/L) × glucose (mg/dL)/405, ≥2.5, was used for the definition of insulin resistance according to previous studies [22,23]. This formula is applicable to glucose concentrations expressed in mg/dL. Moreover, PMOS was classified into the four phenotypes A, B, C and D. The phenotypes can be categorized as follows: phenotype A, comprising all three features of hyperandrogenism, oligo-anovulation and polycystic ovarian morphology (PCOM); phenotype B, including hyperandrogenism and oligo-anovulation; phenotype C, consisting of hyperandrogenism and PCOM; and phenotype D, characterized by oligo-anovulation and PCOM in the absence of hyperandrogenism [24].

### 2.2. Statistical Analysis

Categorical parameters are presented as numbers and frequencies, continuous data as median and their respective interquartile range (IQR). The Mann–Whitney U test was used to compare independent continuous variables. Categorical variables between two groups were compared by chi-square test. Within-group changes were assessed using the Wilcoxon signed-rank test. Between-group differences over time were analyzed using the Mann–Whitney U test. Two-sided Pearson correlations were used, and the correlation coefficients r as well as *p*-values are provided for these analyses. Additionally, because approximately one-third of the study population consisted of women with the PMOS phenotype D, who lack biochemical hyperandrogenism, an exploratory subgroup analysis restricted to phenotypes A, B, and C was performed using the Wilcoxon signed-rank test. Furthermore, an additional post hoc subgroup analysis including only women with baseline hirsutism (FGS ≥ 8) was performed using the Wilcoxon signed-rank test. Given the exploratory nature of this retrospective study, no formal adjustment for multiple comparisons was performed. Consequently, all secondary analyses should be considered exploratory and hypothesis-generating.

Statistical significance was defined by two-sided *p*-values < 0.05. Statistical analyses were performed using SPSS 28.1 (IBM SPSS, USA).

## 3. Results

Both groups’ basic patient characteristics are shown in Table 1. The two groups did not differ in any of the parameters apart from the median modified FGS (DRSP-group: 8, IQR 3–14, versus DYD-group: 5, IQR 2–10; *p* = 0.016). Moreover, there were no differences between the groups concerning the results of hormonal testing at baseline.

The median time interval between treatment initiation and the 3-month follow-up was 97 days (IQR 92–101) in the DRSP-group and 95 days (IQR 91–100) in the DYD-group (*p* = 0.398). Table 2 shows the dynamics of outcome parameters from baseline to three months after treatment. An improvement in the FGS was found only in the DRSP-group (median 8, IQR 3–14, versus median 7, IQR 3–12; *p* < 0.001) but not in the DYD-group (median 5, IQR 2–5, versus median 4, IQR 2–10; *p* = 0.206). At follow-up, in the DRSP-group, serum levels of LH, FSH, testosterone, and the FAI had declined, whereas SHBG had increased (*p* < 0.05). In comparison, in the DYD-group, there was only a significant decline in the FAI. Notably, the DRSP-group revealed lower median LH (5.4 mIU/mL, IQR 3.8–9.5, versus 8.8 mIU/mL, IQR 6.6–13.1; *p* = 0.004), lower median FSH levels (4.5 mIU/mL, IQR 2.9–5.5, versus 5.7 mIU/mL, IQR 4.9–6.3, *p* = 0.001), a lower median FAI (2.9 versus 4.0, *p* = 0.045) as well as higher median SHBG levels (46.4 nmol/L, IQR 39.4–71.9, versus 34.9 nmol/L, IQR 23.1–67.3; *p* = 0.026) at the 3-month follow-up.

One woman (3.3%) stopped the use of the DRSP 4 mg pill due to intermittent bleedings before reaching the 6-month follow-up. No other treatment-related adverse effects were documented in either group during the observation period. Thus, in 29/30 patients (96.7%), a 6-month follow-up after a median of 168 days (IQR 166–172) was available. In the DYD-group, only FGS data were available at 6-month follow-up and this was the case for 26 patients (86.7%). Table 2 provides details. Compared to the 3-month follow-up levels, there was a further decrease in the FGS, in LH levels and the FAI in the DRSP-group at 6-month follow-up (*p* < 0.05). In contrast, the FGS remained stable in the DYD-group.

To better show the effects of DRPS-only treatment on hirsute PMOS patients, we performed a sub-analysis, where only women with a baseline FGS≥ 8 who had received DRSP were included (*n* = 15). Details are shown in Table 3. In these patients, the FGS declined from a median of 14 (IQR 12–16) to 12 (IQR 10–14; *p* < 0.001) after 3 months and then further to 10 (IQR 8–13; *p* = 0.002) after 6 months. Similar patterns to the whole DRSP-group were seen regarding the hormonal values, which included significant declines in the levels of LH, FSH, testosterone and the FAI, whereas SHBG increased (*p* < 0.05). Notably, there was neither a correlation between the baseline- to 6-month dynamics in testosterone levels and in the modified FGS (*r* = 0.060; *p* = 0.832) nor between the dynamics in the FAI and in the modified FGS (*r* = 0.069; *p* = 0.806).

## 4. Discussion

This retrospective cohort study suggests that drospirenone-only treatment may improve clinical hirsutism accompanied by hormonal changes in overweight/obese women with PMOS.

In our study, a high proportion of participants (nearly one-third) were classified as PMOS phenotype D without hyperandrogenemia. This subgroup is unlikely to benefit from antiandrogen therapy. The results in the overall cohort may underestimate the antiandrogenic effect of drospirenone because women without hyperandrogenism were also included. Therefore, we performed a subgroup analysis restricted to PMOS women with hirsutism (FGS ≥ 8). Among affected women receiving DRSP, the median FGS decreased by 4 points after six months of treatment. Notably, DRSP treatment was associated with a significant reduction in FGS after both three months and six months.

Hirsutism, as measured by the modified FGS, warrants particular discussion because its interpretation is the most challenging outcome of the present study. The cosmetic symptoms associated with hyperandrogenism impose a considerable burden on affected women [25]. To date there are limited data about the effects of DRSP-only pills on clinical hyperandrogenism in PMOS patients. In most previous studies, the effect of DRSP in women with PMOS was investigated only in combination with ethinylestradiol (combined oral contraception) [26,27,28]. However, ethinylestradiol contributes substantially to lowering androgen burden by elevating SHBG, which in turn reduces circulating free testosterone [29]. A recent retrospective analysis involving 25 women with PMOS showed a significant improvement in hyperandrogenic symptoms, including acne and hirsutism, following six months of DRSP monotherapy [30]. Comparable to our study, hirsutism was defined using the FGS. The modified FGS decreased from 12.3 to 6.3. In our study, there was also a statistically significant decrease in the DRSP-group. However, in the whole study population, the effect was smaller with a median decrease of only two points in the modified FGS after 6 months (Table 2), whereas the effects seen in the sub-analysis on hirsute women only seem comparable (median decrease of four points, Table 3). Although statistically significant, the two-point reduction in FGS in the overall DRSP-group was modest, and its clinical relevance remains uncertain. The larger four-point reduction observed in women with baseline hirsutism should be interpreted cautiously given the small sample size and exploratory nature of this subgroup analysis.

Notably, in the course of treatment with cyproterone acetate and ethinylestradiol, significant changes in hirsutism are generally seen between 6 and 12 months [31]. With regard to the DRSP-group, following the decreases in FGS and FAI, a further decline was observed at the 6-month follow-up (Table 2). The increase in SHBG and the associated decline in free testosterone/FAI is often seen as one of the main mechanisms for treatment of hirsutism with combined oral contraception [32]. As expected, the DRSP-only pill only led to a minor SHBG increase (Table 2). Thus, the improvement of hirsutism with the DRSP-only pill might be mainly based on its antiandrogenic effects [33]. Thus, one could also compare the effects of DRSP to those of spironolactone on hirsutism. Spironolactone acts as dose-dependent competitive antagonists of the androgen receptor and has additionally been shown to inhibit 5 α-reductase and 17-OH-dehydrogenase activity. Moreover, spironolactone induces both the aromatization of androgens into estradiol and an only minor increase in the hepatic synthesis of SHBG [34,35]. A randomized, double-blind, placebo-controlled trial showed a significant reduction in FGS after six month of 100 mg spironolactone daily with good tolerability [36]. Moreover, in two randomized controlled trials comparing 100 mg of spironolactone with placebo, significant improvements were observed for subjective hair growth (OR 7.18, 95% CI 1.96–26.28) and in the FGS (WMD −7.20, 95% CI −10.98 to −3.42) [37]. Hirsute women with PMOS may benefit from spironolactone treatment, although current evidence is of low certainty and does not demonstrate clear superiority over COCs [38]. Prospective studies comparing DRSP with established antiandrogenic treatments, such as spironolactone, would be valuable to better define its role in the management of hirsutism.

Although it is likely that the improvement of hirsutism is at least in part due to the antagonistic effect of DRSP on the androgen receptor [5], one might hypothesize that it was secondary to a decrease in testosterone levels. Notably, the median testosterone concentration decreased from 0.47 at baseline to 0.42 ng/mL at the 6-month follow-up. (Table 2). At this concentration range, the inter-assay coefficient of variation in the ECLIA method is approximately 5–10%, corresponding to an analytical variation of approximately 0.024–0.047 ng/mL. Thus, although it is statistically significant (*p* < 0.001), the observed difference lies within the expected analytical variability and should not be interpreted as evidence of a clinically meaningful treatment effect.

Together with the mild increase in SHBG levels in the DRSP-group (Table 2), it is reasonable that the FAI decreased in this group. Interestingly, a statistically significant decline in FAI was also observed in the DYD-group. Since dydrogesterone is not known to have antiandrogenic effects or to increase SHBG, this finding should be interpreted cautiously. Hypothetically, this finding could reflect regression to the mean, spontaneous cycle variation, or a type I error in the context of uncorrected multiple testing. Therefore, we do not consider this finding to represent a true pharmacological effect of dydrogesterone. Taking all the above-mentioned considerations together, we consider the reduction in the FAI to also be of only minor clinical relevance for hirsutism. Moreover, neither the dynamics in testosterone nor the dynamics in the FAI showed a significant correlation with the changes in the modified FGS. All in all, we consider that the beneficial effect of drospirenone on skin appearance is attributable less to changes in hormonal status and more to its direct competition mechanism on androgen receptors in the peripheral tissues [39].

Basal LH levels decreased from 10.9 mIU/mL to 5.4 mIU/mL over the 3-month period (*p* < 0.001). After three months of DRSP administration, an adequate suppression of LH could be observed. Reduced levels of LH were expected, as DRSP binds to the progesterone receptor with high progestational potency and exerts a strong antigonadotropic effect, leading to a significant decrease in LH levels [39].

Last but not least, with respect to AMH (Table 2), a slight but significant decline in the DRSP-group was seen (median 7.6 ng/mL versus 7.2 ng/mL, *p* = 0.005) compared to no relevant change in the DYD-group. It should be noted, however, that the use of oral contraceptives is generally associated with a decrease in AMH [40]. Moreover, after three months of therapy, AMH levels remained within a PMOS-specific range [41]. No further decline was observed in the DRSP-group at the 6-month follow-up.

Several limitations should be considered when interpreting these findings. First, only PMOS patients with a BMI ≥25 kg/m^2^ were included. This is due to the fact that the drospirenone-only pill was primarily prescribed to women with metabolic risk factors, whereas the majority of PMOS patients with a lower BMI chose combined oral contraception. Thus, all conclusions drawn from these data have to be limited to overweight/obese PMOS women. Second, the two groups differed significantly in terms of baseline FGS. This baseline imbalance is an inherent consequence of the retrospective open study design, since women in the DYD-group have freely chosen to use cyclical dydrogesterone only and, thus, suffered from hirsutism less often (33.3% versus 50%, Table 1). Since all participants in both groups were of Caucasian ethnicity, differences in ethnicity are unlikely to explain the baseline FGS imbalance. Moreover, it should be noted that the median baseline FGS in the DRSP-group was slightly less pronounced compared to other studies [27,30]. A major limitation of the present study is the potential for selection bias arising from treatment self-selection. As patients were not randomly assigned to therapy but chose or were prescribed their treatment based on clinical characteristics and preferences, this may have influenced baseline differences and the observed treatment effects.

Because dydrogesterone has no known antiandrogenic activity, the present study should not be interpreted as a head-to-head comparison between two antiandrogenic treatments [11,13]. Rather, dydrogesterone served as a clinically relevant neutral control treatment allowing the estimation of changes occurring in women receiving bleeding induction without expected effects on hyperandrogenism. A placebo-controlled prospective study or comparison with an established antiandrogenic treatment would be required to assess the efficacy of DRSP against placebo or active antiandrogen therapy. In addition, the inclusion of a high proportion of women with phenotype D may have attenuated treatment-associated changes in androgen-related outcomes. The subgroup analysis restricted to women with hirsutism was exploratory and not prespecified and should therefore be interpreted with caution. Further prospective studies including only women with hyperandrogenic PMOS are needed to confirm these findings.

Further limitations concern the assessment of hirsutism and androgen levels. No pictures were available for the assessment of the FGS. Patients were instructed, as part of the treatment protocol, to refrain from hair removal for at least two weeks prior to their appointment to enable evaluation of therapeutic outcomes. Moreover, it is known that ethnicity can influence the clinical presentation. In our study, all participants were of Caucasian ethnicity [42]. The use of ECLIA for androgen measurement must be mentioned as a limitation, since liquid chromatography–mass spectrometry is considered the gold standard for testosterone measurement nowadays according to the international guidelines [2].

Finally, the study is clearly limited by its retrospective design and the small sample size. Moreover, the six-month follow-up period limits conclusions regarding the longer-term effects of DRSP, particularly on hirsutism. Due to the retrospective study design, sufficiently complete 12-month follow-up data were not available for analysis. It should also be emphasized that statistical significance does not necessarily reflect clinical relevance. In the present study, several observed changes reached statistical significance but were of small effect size and their clinical impact remains uncertain. Particularly, this refers to the results regarding testosterone levels and FGS, which should therefore be interpreted with caution. Because multiple secondary outcomes were analyzed without adjustment for multiple testing, findings with marginal statistical significance should be interpreted with caution and require confirmation in adequately powered prospective studies [43,44].

Nevertheless, to the best of our knowledge, this is the first comparative study to investigate the antiandrogenic effect of drospirenone monotherapy in PMOS women. We consider our findings promising for further studies in women with hyperandrogenic PMOS. Future prospective studies with clearly defined control groups and randomized designs are warranted to shed more light onto this important topic.

## 5. Conclusions

In conclusion, DRSP-only therapy was associated with modest improvements in clinical and biochemical markers of hyperandrogenism in overweight and obese women with PMOS. Although the overall improvement in hirsutism was modest, a more pronounced reduction was observed in the exploratory subgroup of women with baseline hirsutism. Given the retrospective study design, small sample size, short follow-up period, and the exploratory nature of the subgroup analysis, these findings should be interpreted with caution and require confirmation in adequately powered prospective randomized studies. Nevertheless, DRSP may represent a therapeutic option for hyperandrogenic women with PMOS in whom combined oral contraceptives are not suitable or are contraindicated. By avoiding estrogen exposure, this approach may help to reduce estrogen-related metabolic effects and adverse events.

## Figures and Tables

**Table 1:** Basic patient characteristics of both treatment groups.

		DRSP-Group (n = 30)	DYD-Group (n = 30)	*p*
Age (years) ^1^		31 (29; 34)	31 (29; 34)	1.000
BMI (kg/m^2^) ^1^		27.9 (26.2; 31.5)	28.3 (25.8; 30.2)	0.620
PMOS phenotype ^2^	A	12 (40.0)	11 (36.7)	0.963
	B	5 (16.7)	5 (16.7)	
	C	4 (13.3)	3 (10.0)	
	D	9 (30.0)	11 (36.7)	
FGS ^1^		8 (3; 14)	5 (2; 10)	0.016
FGS ≥ 8 ^2^		15 (50.0)	10 (33.3)	0.295
HOMA-IR ^1^		2.7 (2.3; 3.9)	3.0 (2.3; 4.1)	0.662
HOMA-IR ≥ 2.5 ^2^		19 (63.3)	20 (66.7)	1.000
LH (mIU/mL) ^1^		10.9 (7.4; 13.8)	9.2 (6.3; 14.2)	0.318
FSH (mIU/mL) ^1^		5.3 (4.4; 7.0)	5.7 (5.0; 7.0)	0.270
Testosterone (ng/mL) ^1^		0.47 (0.40; 0.53)	0.47 (0.34; 0.50)	0.325
DHEAS (µg/mL) ^1^		2.85 (1.97; 3.43)	2.25 (1.13; 3.23)	0.271
SHBG (nmol/L) ^1^		40.4 (31.1; 67.2)	31.2 (20.5; 73.6)	0.162
FAI ^1^		3.7 (2.7; 5.8)	5.1 (2.2; 8.2)	0.455
AMH ^1^		7.6 (6.2; 9.4)	7.1 (5.3; 10.5)	0.824

Data are reported as ^1^ median (IQR) for numerical parameters or ^2^ n (%) for categorical parameters. DRSP—drospirenone, DYD—dydrogesterone, BMI—body mass index, PMOS—polyendocrine metabolic ovarian syndrome, FAI—free androgen index, FGS—Ferriman–Gallwey score, HOMA-IR—Homeostatic Model Assessment of Insulin Resistance.

**Table 2:** Differences in hormonal parameters and the modified Ferriman–Gallwey score from baseline to follow-up in both groups.

	DRSP-Group					DYD-Group				
	Baseline (n = 30)	3-Month Follow-Up (n = 30)	6-Month Follow-Up (n = 29)	*p* ^1^	*p* ^2^	Baseline (n = 30)	3-Month Follow-Up (n = 30)	6-Month Follow-Up (n = 29)	*p* ^1^	*p* ^2^
Ferriman–Gallwey score	8 (3; 14)	7 (3; 12)	6 (3; 10)	<0.001	0.001	5 (2; 10)	4 (2; 10)	6 (2; 10)	0.206	0.477
LH (mIU/mL)	10.9 (7.4; 13.8)	5.4 (3.8; 9.5)	4.6 (3.5; 6.6)	<0.001	<0.001	9.2 (6.3; 14.2)	8.8 (6.6; 13.1)	-	0.619	-
FSH (mIU/mL)	5.3 (4.4; 7.0)	4.5 (2.9; 5.5)	4.2 (3.0; 5.2)	<0.001	0.100	5.7 (5.0; 7.0)	5.7 (4.9; 6.3)	-	0.089	-
Testosterone (ng/mL)	0.47 (0.40; 0.53)	0.44 (0.37; 0.53)	0.42 (0.38; 0.52)	0.001	0.092	0.47 (0.34; 0.50)	0.43 (0.38; 0.54)	-	0.297	-
DHEAS (µg/mL)	2.85 (1.97; 3.43)	2.87 (2.00; 3.42)	3.05 (2.20; 3.47)	0.830	0.127	2.25 (1.13; 3.23)	2.05 (1.30; 3.31)	-	0.494	-
SHBG (nmol/L)	40.4 (31.1; 67.2)	46.4 (39.4; 71.9)	48.5 (40.7; 70.4)	0.002	0.737	31.2 (20.5; 73.6)	34.9 (23.1; 67.3)	-	0.198	-
FAI	3.7 (2.7; 5.8)	2.9 (2.3; 3.9)	2.9 (2.3; 3.7)	<0.001	0.023	5.1 (2.2; 8.2)	4.0 (2.5; 7.6)	-	0.045	-
AMH (ng/mL)	7.6 (6.2; 9.4)	7.2 (5.8; 9.2)	6.9 (6.0; 9.1)	0.005	0.178	7.1 (5.3; 10.5)	7.7 (5.2; 9.3)	-	0.127	-

Data are reported as median (IQR). DRSP—drospirenone, LH—luteinizing hormone, FSH—follicle-stimulating hormone, DHEAS—dehydroepiandrosterone sulfate, SHBG—sex hormone-binding globulin, FAI—free androgen index; *p* ^1^—*p*-values comparing the baseline levels to the 3-month follow-up levels; *p* ^2^—*p*-values comparing 3-month follow-up levels to the 6-month follow-up levels.

**Table 3:** Sub-analysis on patients with FGS ≥ 8, who received DRSP (n = 15): Differences in hormonal parameters and the modified Ferriman–Gallwey score from baseline to follow-up.

	Baseline	3-Month Follow-Up	6-Month Follow-Up	*p* ^1^	*p* ^2^
Ferriman–Gallwey score	14 (12; 16)	12 (10; 14)	10 (8; 13)	<0.001	0.002
LH (mIU/mL)	10.1 (7.4; 14.7)	4.2 (3.0; 11.6)	4.5 (3.3; 6.7)	<0.001	0.029
FSH (mIU/mL)	5.2 (4.8; 5.9)	4.7 (2.9; 5.8)	4.2 (3.0; 5.6)	0.017	0.197
Testosterone (ng/mL)	0.51 (0.44; 0.57)	0.47 (0.38; 0.53)	0.45 (0.38; 0.55)	<0.001	0.066
DHEAS (µg/mL)	3.08 (2.37; 3.18)	2.94 (2.31; 3.45)	3.05 (2.36; 3.40)	0.532	0.820
SHBG (nmol/L)	39.5 (26.4; 66.3)	48.9 (33.8; 71.4)	57.4 (39.4; 68.8)	0.004	0.280
FAI	4.2 (3.0; 5.8)	3.0 (2.7; 4.0)	2.9 (2.3; 3.7)	<0.001	0.006

Data are reported as median (IQR). DRSP—drospirenone, LH—luteinizing hormone, FSH—follicle-stimulating hormone, DHEAS—dehydroepiandrosterone sulfate, SHBG—sex hormone-binding globulin, FAI—free androgen index. *p* ^1^—*p*-values comparing baseline levels to 3-month follow-up levels; *p* ^2^—*p*-values comparing 3-month follow-up levels to 6-month follow-up levels.

## Data Availability

The data supporting the findings of this study are available from the authors upon reasonable request.

## Abbreviations

PMOS	Polyendocrine Metabolic Ovarian Syndrome
LH	Luteinizing Hormone
FSH	Follicle-Stimulating Hormone
SHBG	Sex Hormone-Binding Globulin
FAI	Free Androgen Index
AMH	Anti-Müllerian Hormone
DHEAS	Dehydroepiandrosterone Sulfate
BMI	Body Mass Index
FGS	Ferriman–Gallwey Score
DRSP	Drospirenone
DYD	Dydrogesterone
HOMA-IR	Homeostatic Model Assessment of Insulin Resistance
